# Income and Self-Rated Mental Health: Diminished Returns for High Income Black Americans

**DOI:** 10.3390/bs8050050

**Published:** 2018-05-17

**Authors:** Shervin Assari, Lisa M. Lapeyrouse, Harold W. Neighbors

**Affiliations:** 1 Center for Research on Ethnicity, Culture, and Health (CRECH), School of Public Health, University of Michigan, Ann Arbor, MI 48104, USA; 2 Department of Psychiatry, University of Michigan, 4250 Plymouth Rd.; Ann Arbor, MI 48109-2700, USA; 3 Department of Public Health and Health Sciences, University of Michigan, Flint, MI 48502, USA; llapeyro@umflint.edu; 4 Division of Public Health, College of Human Medicine, Michigan State University, Flint, MI 48502, USA; woody.neighbors@hc.msu.edu

**Keywords:** socioeconomic status, socioeconomic position, self-rated mental health, social and economic inequalities, racial and ethnic health disparities, race, African-Americans, Blacks

## Abstract

**Background:** The minorities’ diminished return theory suggests that socioeconomic position (SEP) generates smaller health gains for racial/ethnic minorities compared to Whites. The current study was a Black–White comparison of the association between household income and self-rated mental health (SRMH). **Methods:** This cross-sectional study used data from the 2017 State of the State Survey (SOSS). With representative sampling, the SOSS generates results that are generalizable to the state of Michigan. This study included 881 adults, (*n* = 92) Black and (*n* = 782) White. The independent variable was household income. The dependent variable was SRMH, measured using a single item. Age, gender, and participation in the labor force were covariates. Race/ethnicity was the focal moderator. Logistic regression models were used for data analysis. **Results:** Overall, higher household income was associated with better SRMH, net of covariates. An interaction was found between race/ethnicity and household income on SRMH, suggesting a smaller, or nonexistent, protective effect for Blacks compared to Whites. In race/ethnicity-stratified models, higher household income was associated with better SRMH for Whites but not Blacks. **Conclusion:** Supporting the minorities’ diminished return theory, our study documents differential effects for income on SRHM for Blacks and Whites, where Whites but not Blacks appear to benefit from their income. Given this, researchers and policy makers are cautioned against making assumptions that racial groups benefit equally from similar economic resources.

## 1. Introduction

A large body of research has shown that high socioeconomic position (SEP) promotes populations’ physical and mental health [1,2,3]. The health effects of education [4], employment [5,6], and income [7] against morbidity [8] and mortality [1,2] are well established. High income promotes populations’ self-rated mental health (SRMH) [9,10,11,12] by reducing risk of depression [13], suicide [14,15,16], substance use [9,17].

According to the minorities’ diminished return theory, racial and ethnic minorities do not gain the same health benefits from SEP as White Americans [18,19,20,21,22,23,24,25,26,27]. For example, the protective effect of SEP on physical and mental health is shown to be smaller for Black Americans in comparison to Whites [21,22,27]. Specifically, the protective effects of education [19], employment [23], and income [28] on physical health outcomes are shown to be larger for Whites than Blacks. Despite equal resources across racial groups, the diminished gains experienced by Black Americans are attributed to their disproportionately higher exposure to racism compared to Whites [21,22]. Other structural factors such as residential segregation, higher psychological costs of upward social mobility, as well as interpersonal discrimination may also contribute to the diminished gains of SEP among Blacks compared to Whites [21,22].

Although a protective effect of high SEP against poor mental health is shown by both original studies [29,30,31,32,33,34,35,36,37] and a meta-analysis [9], less is known about the differential effects of income on SRMH across race/ethnic groups. Thus, the purpose of the current study is twofold: (1) to examine whether high SEP is associated with better SRMH and (2) to test for racial variation in the above association. Informed by recent literature on larger mental health gains of SEP indicators for Whites than non-Whites [23,38,39], we hypothesized a weaker association between household income and SRMH for Blacks than for Whites who reside in the State of Michigan.

## 2. Methods

### 2.1. Design and Setting

Using a cross-sectional design, the current study utilizes data from the 2017 State of the State Survey (SOSS). Conducted by the Institute for Public Policy & Social Research (IPPSR), Michigan State University (MSU), SOSS is the only survey conducted in Michigan designed to systematically monitor social, political, and economic attitudes and beliefs in major regions throughout the state [40].

The SOSS is a telephone survey that includes a random sample of approximately 1000 Michigan residents. The survey takes approximately 20 min to complete. The first seven minutes of the telephone survey collect basic demographic information and monitors the public’s satisfaction with existing economic conditions [40].

The SOSS enrolls participants using a stratified random sample of adults (i.e., age 18 and older) living in the state of Michigan. The SOSS data include “weights” that should be adjusted so the results are representative of the adult population of Michigan residents [40]. 

### 2.2. Sampling Eligibility

The eligibility criteria for the SOSS include: (1) age 18 and over; (2) residence in Michigan; (3) being non-institutionalized; and (4) being English-speaking. As SOSS is a telephone survey, only adults who lived in a household with a landline telephone or individuals with a Michigan cell phone number could be interviewed [41].

### 2.3. Sampling Procedure

The SOSS sample is comprised of both new and old participants. Usually, 60–80% of the sample are new participants who are interviewed for the first time. This sample is drawn from a newly generated list of random-digit-dial (RDD) phone numbers for Michigan. The remainder of the SOSS sample is comprised of previous SOSS participants. This proportion of the sample includes participants who had been interviewed two years prior (i.e.; 2015 participants who agree to be re-interviewed). In general, 80–90% of all SOSS respondents agree to be re-contacted. Both sub-samples constitute a representative random sample. Given that many households have opted not to have a landline telephone, in favor of cell phones, the SOSS includes a sample of cell phone users. The SOSS sampling frame is a sample of randomly generated telephone numbers (landline or cell phone) provided by the Survey Sampling, Inc. (SSI; https://www.surveysampling.com) [41].

For the 2017 SOSS sample, 12,007 phone numbers were used. From this number, 584 were in the re-contact segment, 5897 were in the new RDD segment, and 6500 were in the new cell phone segment. Overall, 48.2% of phone numbers were working (79.8% for the re-contact segment, 50.2% for the new RDD segment, and 43.6% for the new cell phone segment) [41].

### 2.4. Data Collection

Data were collected by the of IPPSR’s Office for Survey Research (OSR). Interviews were conducted between 19 April and 30 July 2017. All interviews used a computer-assisted telephone interviewing system (CATI). In CATI, interviews are scripted and executed from a computer workstation. During the interview, the questions and the instructions are provided for the interviewers on their computer screens. The computer also indicates what numeric codes or text can be potentially entered as responses to each item. For interviews, the Computer Assisted Survey Execution System (CASES, version 5.5) software was used. CASES is collectively developed by the U.S. Department of Agriculture, the U.S. Census Bureau, and University of California—Berkeley [41].

### 2.5. Interviewer Training

Approximately 38 people were trained to collect the 2017 SOSS data. Interviewers received training regarding the study protocols, the interview instrument, and the objectives of the various questions. Experienced interviewers received two hours of study-specific training. New interviewers were given 13 training hours of training, including one shift of practice interviewing. All the interviewer trainees received training manuals with instructional materials such as procedures, forms, and a full description of all operations [41].

### 2.6. Ethical Considerations

The study was approved by the Institutional Review Board of the Michigan State University (MSU). Informed consent was obtained from all participants (Appendix A). All SOSS participants received financial compensation for their time.

### 2.7. Measures

#### 2.7.1. Independent Variable

Household income was the primary independent variable in this study. Household income was self-reported. Household Income was composed of the following 11 categories: (1) below $10,000; (2) $10,000–$20,000; (3) $20,000–$30,000; (4) $30,000–$40,000; (5) $40,000–$50,000; (6) $50,000–$60,000; (7) $60,000–$70,000; (8) $70,000–$90,000; (9) $90,000–$100,000; (10) $10,000–$150,000; (11) above $150,000. Income was operationalized as a continuous measure.

#### 2.7.2. Dependent Variable

The outcome variable of interest was self-rated mental health (SRMH). Participants were asked “How would you rate your overall mental health?” Responses included five categories: (1) excellent; (2) very good; (3) good; (4) fair; and (5) poor. Single-item SRMH measure correlates with psychiatric disorders and mental health [42]. Test-retest reliability of SRMH is 0.7–0.8 [42]. Single-item self-rated health measures are repeatedly used across racial groups [43]. In this study, we dichotomized SRMH as poor/fair = 1 versus excellent to good = 0.

#### 2.7.3. Covariates

Sociodemographic covariates in the current study included age, gender, labor market participation, and education. Age was operationalized as a continuous variable. Gender was a dichotomous variable (male 1 vs. female 0). Education was measured as a dichotomous variable: (1) has not completed college; (2) completed college. Employment was measured as a dichotomous variable (non-participation vs. participation) in the labor market. 

#### 2.7.4. Moderator

Self-identified race/ethnicity was measured. Race/ethnicity was treated as a dichotomous variable with Whites as the reference category (Blacks = 1 vs. Whites = 0).

### 2.8. Statistical Analysis

Using 5-year estimates from the 2010–2014 American Community Survey, SOSS participants are weighted so that the proportions of Whites and Blacks in the sample are equivalent to the White and Black adult population of Michigan. 

We applied the SOSS sampling weights using Stata 13.0 (Stata Corp.; College Station, TX, USA) to perform all the data analyses. Thus, the results presented here are representative of Michigan residents (adults). For our analyses, design-based standard errors were estimated using Taylor series linearization. As we were only interested in Whites and Blacks in this study, we used sub-population survey commands that accommodate subsample analysis.

For descriptive purposes, we used mean (SE) and proportions in the overall sample and race/ethnicity. For bivariate analyses, we applied a Pearson correlation test, independent sample *t* test, and Pearson Chi-square. For multivariable analysis, we used logistic regression models. From logistic regression models, adjusted odds ratios (OR), 95% confidence intervals (CI), and associated *p* value levels are reported. 

We ran four logistic regression models in which household income was the independent variable, poor/fair SRMH was the dependent variable, and age, gender, education, and labor market participation were the control variables. The first two logistic regression models were estimated in the overall sample while the last two models examined relationships by race/ethnicity. *Model 1* did not explore the moderating effect of race/ethnicity and income on SRMH and thus did not include an interaction term; however, *Model 2* included the race/ethnicity by household income interaction term. *Model 3* was estimated for Whites and *Model 4* was calculated for Blacks.

## 3. Results

### 3.1. Descriptive Statistics

Table 1 provides descriptive statistics in the overall sample as well as by race/ethnicity. Blacks had lower education, employment, and household income compared to Whites. Blacks also had worse SRMH than Whites.

### 3.2. Bivariate Correlations

Table 2 summarizes the bivariate correlations between study variables in the overall sample. Age, education and household income showed negative correlations with poor SRMH.

### 3.3. Logistic Regressions in the Overall Sample

Table 3 summarizes the results of two logistic regressions in the overall sample. Both models had household income as the independent variable, poor SRMH as the dependent variable, and age, gender, employment, and education as covariates. *Model 1* only included the main effects. *Model 2* also included the race/ethnicity by household income interaction term. *Model 1* showed an inverse association between household income and odds of poor SRMH above and beyond the covariates. *Model 2* also showed a significant interaction between race/ethnicity and household income on poor SRMH, indicating smaller protective effects of household income against poor SRMH for Blacks than Whites. 

### 3.4. Logistic Regressions by Race/Ethnicity

**Figure 1:**
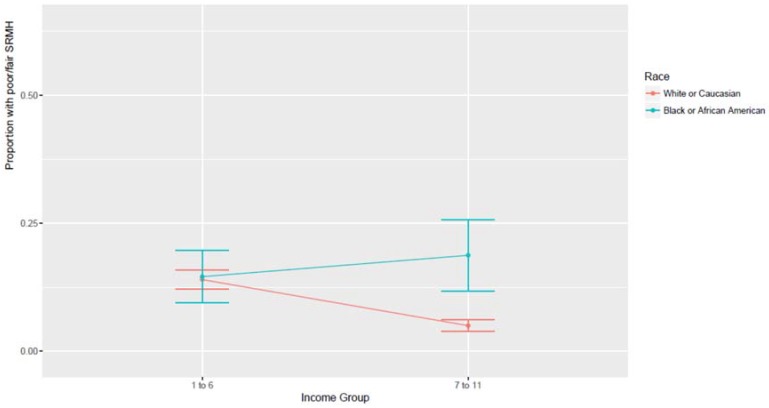
Association between income and SRMH by race.

Table 4 provides a summary of two additional logistic regression models which were estimated specifically for Whites and Blacks. *Model 3* showed that in Whites, higher household income was associated with lower odds of poor SRMH. *Model 4* showed that in Blacks, higher household income was not associated with lower odds of poor SRMH. Figure 1 shows the association between income and SRMH by race/ethnicity. As this figure shows, the largest Black–White difference in SRMH exists at the highest income levels, and Blacks and Whites are no different for low levels of income. 

## 4. Discussion

The current study had two aims: first, to test whether high SEP is associated with lower risk of poor SRMH, and second, to explore racial variation in the association between household income and poor SRMH. Our first finding showed that higher household income is associated with better SRMH overall. Our second finding showed that this mental health gain is smaller for Blacks than for Whites. 

The first finding on the overall association between higher household income and better SRMH is consistent with the fundamental cause [44,45] and social determinants of health [46,47] theories that consider low SEP as root cause of poor health. This finding is also supported by a considerable amount of empirical evidence documenting the link between high SEP and better SRMH [10,11,12]. In addition to SRMH, higher income protects people against depression [13], suicide [14,15,16], and substance use [9,17]. The protective effects of income on mental health seem to be non-specific as the health benefits of higher income extend to multiple outcomes [48]. 

This is not the first study to show that race/ethnicity alters the protective health effect of SEP. Most of this literature, however, has focused on physical rather than mental health outcomes [49,50,51,52,53]. The current study makes an important contribution to the literature on the differential mental health effects of SEP by race/ethnicity by extending the minorities’ diminished return theory [21,22] with empirical evidence on how race/ethnicity modifies the impact of higher income on self-reported mental health. 

These results are consistent with the minorities’ diminished return theory [21,22], which has documented a smaller health return of high SEP for Blacks compared to Whites [19,20,23]. Household income reduces chronic medical conditions significantly more for Whites than Blacks [23], which has been attributed to such factors as the racial segregation of Blacks to communities where access to health resources are limited and more costly, thus diminishing Blacks’ purchasing power [54]. Employment also protects health disproportionately more for Whites than Blacks [23], as the types of jobs held by Blacks are more likely to expose them to health hazardous work conditions, while also failing to offer employee sponsored health insurance [23,55]. Further, education is more strongly related to impulse control [56], drinking [20], smoking [57], diet [58], self-rated health [59], and oral health [60] for Whites than Blacks. These may, in part, be explained by the fact that education generates higher income for White compared to Black families [61].

The smaller effects of lower income on the mental health of Blacks may be due to systemic resilience or system blaming of Blacks who live under adversity [62,63]. This view is supported by studies showing equal mental health outcomes of low and high-income Blacks [64]. Poor Blacks’ high mental health may also be a result of thriving and flourishing under economic adversities [65,66]. In a unique study of 34 Black female, 32 Black male, 31 White female, and 37 White male upper- and middle-class adults, Steele found an interaction between race and direction of social mobility on mental health; upwardly-mobile Blacks and downwardly-mobile Whites felt more self-critical [67]. 

The results are relevant to a literature that has documented poorer mental health among high SEP Blacks compared to their low SEP counterparts [18,68]. However, more information is available on depression than SRMH [26,38]. Studies have found higher risk of depression in high income Black boys [38] and men [26] compared to their lower income counterparts. Another study showed that Black men with higher educational attainment were the only race by gender group that showed an increase in their depressive symptoms over time [18]. These findings were partially attributed to the higher risk of discrimination in high SEP Blacks [24,69].

Not only economic resources but psychological assets such as affect, coping, sleep, and self-efficacy may better serve the mental health of Whites than Blacks [70,71,72,73,74,75,76,77,78]. As these psychological constructs are mediators for the effects of SEP on health, their differential effects by race may explain our finding on racial variation in the effects of SEP on SRMH. Future research should test whether coping and other psychosocial factors explain racial variation in the SEP-SRMH link. 

The minorities’ diminished return theory attributes the diminished return of SEP for Blacks to racism, discrimination, and the unfair treatment of Blacks in the United States [21,22]. One-hundred and fifty-five years after slavery was abolished, and decades after the Civil Rights Movement, structural racism remains embedded in many aspects of the U.S. U.S. cultural values emphasize the importance of individual responsibility, which often ignores societal barriers that are more common in the lives of Blacks. Through the lens of American individualism, structural factors that disproportionately affect Blacks are systemically overlooked by policy makers [79,80]. Without a dramatic change to the social structure of the United States, policy-makers will continue to maintain White privilege, which is a significant determinant of the diminished mental health returns for Black Americans [21,22].

These effects of racism and racial discrimination have biological implications [81,82]. Racism, racial discrimination, and the resulting trauma not only damage health over the course of life; they also result in transgenerational harm. There is evidence to suggest that the chronic stress to which Black Americans are exposed has lasting effects on telomere length, a biomarker for physiologic damage. Specifically, shortened telomere length may play a significant role in the diminished health returns experienced by Black Americans. Chae, et al. [81], and several other scholars [82,83,84,85,86] have all discussed the physiologic damaging effects of prolonged, chronic stress. Geronimus et al. propose the “weathering” process and its age-related patterns for allosteric load scores [87]. Although these explanatory models have differences, they share an emphasis on how adversity, trauma, and stress can result in health consequences at the biological level. The combination of exposure to race-based chronic stress, biopsychosocial coping responses, and structural barriers to medical treatment can have trans-generational consequences together, especially through mechanisms (e.g., fetal exposure to maternal stress) related to the health of pregnant Black women [88,89]. 

The minorities’ diminished return theory does not attribute the smaller mental health gains of higher SEP for Blacks as a consequence of mismanaged economic resources. Nor does this theory argue that Whites are more effective in using financial resources. Rather, the minorities’ diminished return theory focuses on the inability of the current U.S. opportunity structure (i.e., education, employment, housing, etc.) to provide an equitable distribution of benefits across race. Racial discrimination results in additional structural barriers Blacks must overcome in order to achieve the typical mental health benefits of improved socioeconomic position. The persistent and robust nature of racial health disparities makes this abundantly clear—racial/ethnic health disparities are not reduced or eliminated by SEP. In general, Black Americans display higher rates of morbidity and mortality at every level of socioeconomic position. This means that high SEP Blacks who have successfully climbed the social ladder, earning high incomes, are typically in poorer health than similarly situated Whites. Indeed, U.S. society may make upper-status Blacks pay extra psychological costs for upward social mobility. For instance, high SEP Blacks may have higher goal striving stress or John Henryism than low SEP Blacks that increase their risk of poorer physical and mental health [90,91,92,93,94,95,96].

The current study has implications for theory. Most of the research on disparities has focused on differential exposure rather than the combination of exposure and differential response [21,22]. As a result, the contribution of differential gain from similar resources as a mechanism behind health disparities is traditionally overlooked [19,27]. Researchers working on Black–White differences should systemically explore interactions among race/ethnicity, financial, personal, and social resources as determinants of mental health [21,22]. The results of this study demonstrate that it is Whites, not Blacks, whose mental health is more closely associated with SEP indicators. This finding is in contrast to the theories such as double jeopardy [38], triple jeopardy [97], multiple jeopardy [98] and multiple disadvantage [99]. All of these theories conceptualize racial and ethnic minorities as vulnerable populations, assuming that their mental health outcomes will be more strongly affected by the absence or presence of any additional risk factor, compared to Whites [98]. 

### Limitations

This study is not without limitations. Participants were limited to a sample with landlines and local cellphones, and the study excluded non-English speakers. Given the cross-sectional design of our study, we are unable to establish causal associations. SEP indicators such as household income and mental health have reciprocal associations. Further, changes in SEP over the life course have important implications for understanding the SES-health relationship as health assaults encountered early in one’s life are found to take a significant toll on the health of upwardly mobile Blacks [100]. Future research should use longitudinal data to rule out reverse causality between poor mental health and household income as well as explore how changes is SEP over one’s life course contribute to between and within group differences in mental health among Blacks and Whites. This is particularly important as the downwardly-mobile Blacks may have a different mental well-being than persistently poor Blacks [62]. 

Another limitation of the study was unmeasured confounders. We did not include several confounding factors such health insurance, poverty status, or physical health. Our outcome (SRMH) was also a single item measure. Future research should replicate these findings using more comprehensive measures such as psychiatric disorders, mental health care use, mental health care need, and mental health care costs. Differential validity may also be a threat to the current study. SRMH may have different meanings and poor SRMH may reflect different health problems in Whites and Blacks [43]. All the variables in the current study were limited to individual characteristics. Thus, future research should include contextual measures such as high-level SES, urbanity, the availability of resources, community safety, social capital, and density of Whites and Blacks in the community, as these factors have also been shown to impact mental health [101,102,103]. More research is also needed on the non-significant relationship of income to SRMH for Blacks. There is a need to replicate this finding in other data sets, particularly those with a national sample. 

## 5. Conclusions

While household income is associated with better mental health, the magnitude of this association depends on race/ethnicity. Additional research is needed to better understand the conditional associations among SEP, race/ethnicity, and SRMH. Policies are also needed to narrow Blacks’ diminished returns.

## Figures and Tables

**Table 1:** Descriptive statistics in the pooled sample and by race.

Characteristics	All		Whites		Blacks	
	%	95% CI	%	95% CI	%	95% CI
Race						
Whites	84.76	80.94–87.93				
Blacks	15.24	12.07–19.06				
Gender						
Women	52.80	48.72–56.84	51.98	47.75–56.18	57.34	44.42–69.33
Men	47.20	43.16–51.28	48.02	43.82–52.25	42.66	30.67–55.58
Education (≥12 years) *^a^						
Less than college	55.46	51.36–59.49	52.47	48.22–56.69	72.08	59.22–82.10
Completed college	44.54	40.51–48.64	47.53	43.31–51.78	27.92	17.90–40.78
Employment *^a^						
Not in labor force	36.59	32.89–40.45	38.62	34.68–42.72	25.28	16.71–36.31
In labor force	63.41	59.55–67.11	61.38	57.28–65.32	74.72	63.69–83.29
SRMH *^a^						
Good–excellent	88.22	85.06–90.78	90.03	87.21–92.29	78.11	64.35–87.58
Poor/Fair	11.78	9.22–14.94	9.97	7.71–12.79	21.89	12.42–35.65
	**Mean**	**95% CI**	**Mean**	**95% CI**	**Mean**	**95% CI**
Age (years) *	48.24	46.63–49.86	50.25	48.57–51.94	43.48	38.17–48.79
Household income (USD10,000) *^b^	6.60	6.33–6.86	6.70	6.43–6.97	5.54	4.62–6.47

* *p* < 0.05 for comparisons of Whites and Blacks. ^a^ Pearson Chi square; ^b^ independent samples *t* test. Source: State of the State Survey (SOSS), 2017.

**Table 2:** Spearman correlations in the pooled sample and by race.

Characteristics	1	2	3	4	5	6	7
1 Race/ethnicity (Black)	1.00						
2 Gender (men)	−0.07	1.00					
3 Age	−0.16 *	−0.05	1.00				
4 Employment (in labor force)	0.06	0.09 *	−0.46 *	1.00			
5 Education (completed college)	−0.09 *	0.01	−0.01	0.11 *	1.00		
6 Household Iincome	−0.09 *	0.16 *	−0.14 *	0.29 *	0.37 *	1.00	
7 Poor SRMH	0.05	−0.07	−0.10 *	−0.04	−0.11 *	−0.15 *	1.00

* *p* < 0.05; Source: State of the State Survey (SOSS), 2017. SRMH: self-rated mental health.

**Table 3:** Summary of logistic regressions between household income and poor SRMH in the pooled sample.

Characteristics	*Model 1* Main Effects		*Model 2* Model 1 + Interactions	
	B	95% CI	B	95% CI
Race/ethnicity (Black)	1.93	0.87–4.30	0.48	0.09–2.53
Gender (men)	0.80	0.43–1.48	0.76	0.41–1.41
Age	0.96 *	0.95–0.98	0.96 *	0.95–0.98
Employment (in labor force)	0.58	0.28–1.22	0.59	0.29–1.20
Education (Completed college)	0.57	0.28–1.14	0.59	0.30–1.18
Household income	0.88 **	0.78–0.99	0.82 *	0.73–0.92
Household income * race/ethnicity		--	1.29 *	1.01-1.66

* *p* < 0.05, ** *p* < 0.01. Outcome: poor self-rated mental health (SRMH). Source: State of the State Survey (SOSS), 2017.

**Table 4:** Summary of logistic regressions between household income and poor SRMH in Whites and Blacks.

Characteristics	*Model 3* Whites		*Model 4* Blacks	
	B	95% CI	B	95% CI
Gender (men)	0.55 ^#^	0.28–1.09	1.14	0.14–9.01
Age	0.98 *	0.96–1.00	0.89 **	0.83–0.95
Employment (in labor force)	0.72	0.35–1.48	0.27	0.02–3.87
Education (completed college)	0.89	0.44–1.81	0.08 ^#^	0.01–1.34
Household income	0.81 **	0.72–0.90	1.10	0.74–1.62

^#^*p* < 0.1, * *p* < 0.05, ** *p* < 0.001. Outcome: poor SRMH. Source: State of the State Survey (SOSS), 2017.

## Appendix A. State of the State Survey (SOSS) Consent

Before we begin, let me tell you that this interview is completely voluntary. You may choose not to participate and you may end your participation at any time without penalty. Should we come to any question that makes you feel too uncomfortable or you do not want to answer, just let me know and we can go on to the next question. Information collected for this study will be kept confidential to the extent allowed by local, state and federal law, and no reference will be made in any oral or written report that would link you individually to this study. If you have any questions about this study or would like contact information for the investigators, you can ask me at any time. This call may be recorded or monitored for quality assurance.

## Appendix B. State of the State Survey (SOSS) Regions

1. Upper Peninsula: Alger, Baraga, Chippewa, Delta, Dickinson, Gogebic, Houghton, Iron, Keweenaw, Luce, Ontonagon, Mackinac, Marquette, Menominee, Schoolcraft
2. Northern Lower Peninsula: Alcona, Alpena, Antrim, Benzie, Charlevoix, Cheboygan, Crawford, Emmet, Grand Traverse, Iosco, Kalkaska, Leelanau, Missaukee, Montmorency, Ogemaw, Oscoda, Otsego, Presque Isle, Roscommon, Wexford
3. West Central: Allegan, Barry, Ionia, Kent, Lake, Manistee, Mason, Mecosta, Montcalm, Muskegon, Newaygo, Oceana, Osceola, Ottawa
4. East Central: Arenac, Bay, Clare, Clinton, Gladwin, Gratiot, Huron, Isabella, Midland, Saginaw, Sanilac, Shiawassee, Tuscola
5. Southwest: Berrien, Branch, Calhoun, Cass, Eaton, Hillsdale, Ingham, Jackson, Kalamazoo, St. Joseph, Van Buren
6. Southeast: Genesee, Lapeer, Lenawee, Livingston, Macomb, Monroe, Oakland, St. Clair, Washtenaw, Wayne [excluding Detroit]
7. Detroit
